# Prevalence and clinical significance of the genotypic carriage among ESBL phenotype-negative *Escherichia coli* and *Klebsiella pneumoniae* clinical isolates in bacteremia: a study in a Malaysian tertiary center

**DOI:** 10.3389/fcimb.2024.1429830

**Published:** 2024-10-24

**Authors:** Chee Lan Lau, Hui-min Neoh, Petrick Periyasamy, Tg Mohd Ikhwan Tg Abu Bakar Sidik, Toh Leong Tan, Ramliza Ramli, Isa Naina Mohamed

**Affiliations:** ^1^ Pharmacoepidemiology and Drug Safety Unit, Department of Pharmacology, Faculty of Medicine, National University of Malaysia, Kuala Lumpur, Malaysia; ^2^ Pharmacy Department, Hospital Canselor Tuanku Muhriz, Kuala Lumpur, Malaysia; ^3^ UKM Medical Molecular Biology Institute (UMBI), National University of Malaysia, Kuala Lumpur, Malaysia; ^4^ Faculty of Health Sciences, National University of Malaysia, Kuala Lumpur, Malaysia; ^5^ Medical Department, Faculty of Medicine, National University of Malaysia, Kuala Lumpur, Malaysia; ^6^ Emergency Medicine Department, Faculty of Medicine, National University of Malaysia, Kuala Lumpur, Malaysia; ^7^ Department of Medical Microbiology and Immunology, Faculty of Medicine, National University of Malaysia, Kuala Lumpur, Malaysia

**Keywords:** *Escherichia coli*, *Klebsiella*, ESBL, bacteremia, antibiotic resistance, polymerase chain reaction

## Abstract

**Background:**

Antimicrobial resistance (AMR) can lead to fatal consequences. AMR genes carriage by phenotypically susceptible bacteria, such as Extended-Spectrum β-Lactamases (ESBL)s in *Enterobacteriaceae*, have potential implications for AMR spread and therapeutic outcomes. This phenomenon should be investigated.

**Methods:**

Positive blood cultures from hospitalized patients in a Malaysian tertiary center between April 2022 and March 2023 were reviewed. A total of 137 clinical isolates of *Escherichia coli (E.coli*), *Klebsiella pneumoniae* (*K.pneumoniae*), and *Klebsiella oxytoca* were included. The antibiotic susceptibility and ESBL phenotypes were determined by disk diffusion method and the identification of genotypes by multiplex polymerase chain reaction. The clinical characteristics and outcome information were extracted by reviewing patients’ medical records to evaluate the clinical significance of the ESBL genotype-positive but phenotype-negative isolates in bacteremia.

**Results:**

All 137 isolates were positive for at least one genotype (*bla*_CTX-M_, n = 71, 51.8%; *bla*_SHV_, n = 87, 63.5%; *bla*_TEM_, n = 95, 69.3%; *bla*_OXA-1_, n = 38, 27.7%). While *bla*_CTX-M_ was proportionately higher in the ESBL phenotype-positive isolates than ESBL phenotype-negative isolates (33/37, 89.2% vs 38/100, 38%; p < 0.001), more than half of those harboring *bla*_CTX-M_ remained susceptible to third-generation cephalosporins (3GC). The sensitivity (Sen) of *bla*_CTX-M_ for ESBL phenotypes prediction was 89.19% (95% confidence interval [CI], 74.58 - 96.97%); however, specificity (Sp) was low (46.47%; 95% CI 39.75 - 53.32). The patient characteristics were similar among 98 ESBL phenotype-negative cases, except that the non-*bla*_CTX-M_ carrier group had significantly more renal impairment (0/37 vs 7/61, p = 0.043) and gastrointestinal sources of bacteremia (9/37 vs 27/61, p = 0.047). No differences were observed in infection severity, in-hospital mortality, and length of stay (LOS) between the *bla*_CTX-M_ and non-*bla*_CTX-M_ carrier groups.

**Conclusion:**

The current study provides insight into the gene carriage in *E.coli* and *Klebsiella species* clinical isolates, including *bla*_CTX-M_ genotypes in antibiotic-susceptible strains from a Malaysian hospital. The ESBL encoding genotypes such as *bla*_CTX-M_ presented substantially beyond one-third of the ESBL phenotype-negative or 3GC susceptible *E.coli* and *K.pneumoniae* isolated from bloodstream infection. Although clinical outcomes were not worsened with *bla*_CTX-M_ genotype-positive but ESBL phenotype-negative isolates in bacteremia, the potential implications for AMR spread deserve further investigation.

## 1 Introduction

Antimicrobial resistance (AMR) is part of the natural process for the evolution and survival of microorganisms. However, the AMR development and distribution rate is accelerated via the selection pressure exerted by antimicrobials (Davies and Davies, 2010). AMR can lead to fatal consequences. The first global report predicted that the number of deaths attributed to AMR could strike 10 million, inclusive of the 4 million in Asian countries, by the year 2050 (World Health Organization, 2016). Concurring with the WHO priority pathogen list (World Health Organization, 2017), the global assessment by Murray et al. (2022) identified *Escherichia coli (E.coli*), *Klebsiella pneumoniae* (*K.pneumoniae*), in addition to third-generation cephalosporins (3GC) resistance and bacteremia, as the primary causative agents of AMR-related mortality. Furthermore, increasing *K. pneumoniae* 3GC resistance in bacteremia reported in the recent Global Antimicrobial Resistance and Use Surveillance System (GLASS) report urges immediate attention, together with *E.coli* as the standard indicator organism for AMR monitoring (World Health Organization, 2022). Ascending resistance rates pose a pressing concern about treatment adequacy and highlight the need for diagnostic technology advancement in AMR pathogen identification and tracking.

Extended-spectrum β-lactamases (ESBL)s are the most common resistance mechanism among *Enterobacteriaceae* (Noster et al., 2021). *bla*_CTX-M_ genes are the most prevalent genes encoding ESBL in *Enterobacteriaceae*, followed by *bla*_TEM_ and *bla*_SHV_ (Tamma et al., 2022). *bla*_CTX-M_-encoding enzymes could inherently hydrolyze extended-spectrum cephalosporins (such as ceftriaxone, cefotaxime and ceftazidime) and also β-lactam/β-lactam inhibitors (such as piperacillin/tazobactam) upon mutation (De Angelis et al., 2020a). Genotypic testing of β-lactamases genes in clinical isolates was proposed to picture the ESBL prevalence better and unravel the potential silent spread (Tamma and Humphries, 2021). However, most of the prevalence studies of ESBL genes were assessed in ESBL phenotype-positive or 3GC-resistant strains (Ling et al., 2021; Tamma et al., 2021). Limited studies investigated the carriage of ESBL genes in non-ESBL phenotype isolates (Tamma and Humphries, 2021; Deekshit and Srikumar, 2022). The presence of unexpressed genes in antibiotic susceptible bacteria is not uncommon (Yee et al., 2021), but little is known about the association with the implication to clinical settings and public health (Deekshit and Srikumar, 2022). Interestingly, only sporadic studies from Asian countries (Zhang et al., 2018; Son et al., 2021) revealed the presence of ESBL genes, such as *bla*_CTX-M,_ among non-ESBL phenotype isolates. The study in Vietnam by Son et al. (2021) detected *bla*_CTX-M_ in nearly half of ESBL phenotypes-negative isolates and observed higher mortality and prolonged hospitalization than those without *bla*_CTX-M_. This resulted in apprehension regarding the magnitude of unanticipated resistance and the possible correlation with adverse clinical outcomes (Lee et al., 2015; Son et al., 2021).

Malaysia is one of the developing countries with a worrying incidence of AMR since the early 90s. A recent systematic review found Malaysia has the second highest ESBL-producing *K.pneumoniae* prevalence (76%) among Southeast Asia countries (Salawudeen et al., 2023). Following the establishment of the national antibiotic resistance surveillance program, the prevalence of ESBL-producing *Enterobacteriaceae* in the country is rising (Iskandar et al., 2021). The latest national surveillance reported that the resistance rate against cefotaxime among blood isolates of *E.coli* was 27.5%, while that of *K.pneumoniae* was 34.8% (IMR, 2023). Furthermore, local studies revealed that the mortality rate associated with ESBL phenotype-negative *K. pneumoniae* in bacteremia was 12.3% (Ang et al., 2022) and soared to 30.2% among ESBL phenotype-positive isolates (Hazwan et al., 2022). Earlier studies in Malaysia showed that *bla*_TEM_ and *bla*_CTX-M_ predominated in clinical isolates of ESBL-producing *E.coli* from various body sites (Sekawi et al., 2008; Lim et al., 2009a; Othman et al., 2016), whereas ESBL-producing *K.pneumoniae* frequently carried *bla*_SHV_ (Lim et al., 2009b). Of *bla*_CTX-M_ genes, *bla*_CTX-M_-_1_ and *bla*_CTX-M_-_9_ were the dominant genotypes in Malaysian studies (Othman et al., 2016; Ngoi et al., 2021). However, most genotyping studies (including those in Malaysia) involve ESBL phenotype-positive isolates (Dwiyanto et al., 2020; Ngoi et al., 2021; Yap et al., 2023). To better understand the attributes of β-lactamases to rising AMR and clinical implication, genotypic analysis should be extended to include susceptible or ESBL phenotype-negative isolates (Stasiak et al., 2021; Deekshit and Srikumar, 2022).

In this study, we investigated the presence of genotypes among *E. coli* and *Klebsiella species* (*K.pneumoniae* and *Klebsiella oxytoca* [*K.oxytoca*]) clinical isolates in bacteremia. We further examined the correlation of *bla*_CTX-M_ with ESBL phenotypes and the clinical significance among the ESBL genotype-positive but phenotype-negative isolates.

## 2 Materials and methods

### 2.1 Study setting and population

This cross-sectional study was conducted at the Hospital Canselor Tuanku Muhriz, National University of Malaysia (HCTM, UKM), a 1054-bed tertiary care hospital. Microbiological investigations were carried out by the in-house HCTM UKM’s diagnostic laboratory services. Blood cultures from hospitalized patients were sent as part of routine clinical care and incubated in a BD BACTEC TM Fx system (BD, Franklin Lakes, NJ). Organisms were identified by an automated VITEK^®^ 2 system (bioMérieux, Marcy-l’Etoile, France) or matrix-assisted laser desorption ionization-time of flight mass spectrometry analysis (MALDI-TOF MS) (Bruker Daltonics, Bremen, Germany). For bacteria identification using MALDI-TOF MS, direct colony extraction technique was used by smearing the fresh colony material as a thin film onto a spot on a MALDI 96 target plate (Bruker Daltonik) and overlaying with one μL of a saturated a-cyano-4-hydroxy-cinnamic acid (HCCA) matrix solution in 50% acetonitrile-2.5% trifluoroacetic acid (Bruker Daltonik) and allowing to dry at room temperature before MS analysis. Alternatively, extended direct transfer was used by overlaying the smeared spot with one μL 70% formic acid and allowing it to dry at room temperature before overlaying with the HCCA matrix. Mass Spectrum analyses were referred to the database provided in MALDI BIOTYPER (Bruker Daltonics, Bremen, Germany), software version compass 4.1.100 containing library version 12 and library number 11897. The definite species identification of bacteria was based on the score value of >/= 1.7.

**Figure 1:**
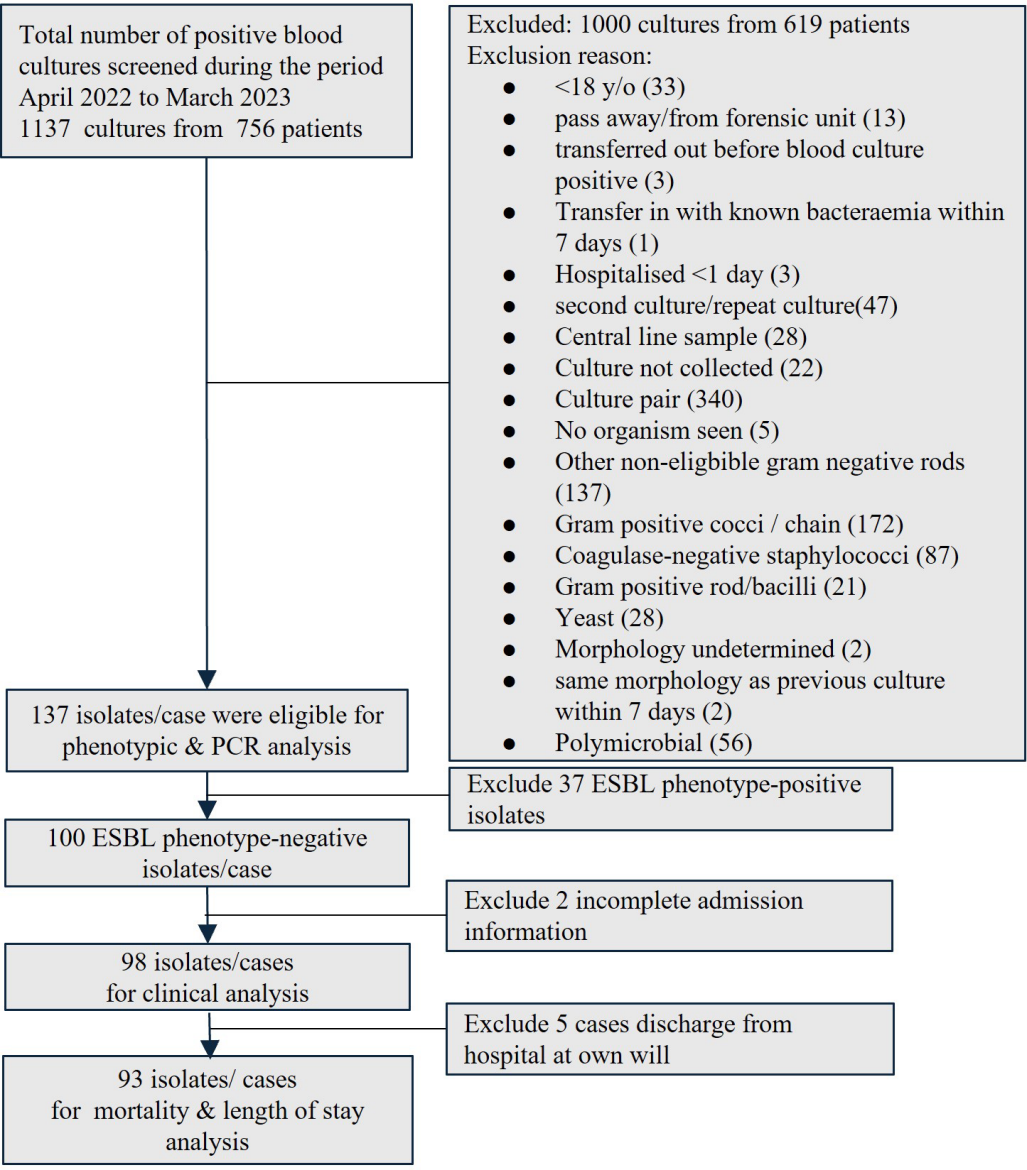
Enrollment of blood isolates and patients into the study. (ESBL, extended-spectrum β-lactamase; PCR, polymerase chain reaction).

The positive blood cultures from patients aged > 18 years old between April 2022 and March 2023 were screened for positive mono-bacterial cultures of *E.coli*, *K.pneumoniae*, and *K.oxytoca*. The corresponding blood agars with colonies were collected from in-house HCTM UKM’s diagnostic laboratory. Patient unique identifiers were removed, and each sample was given a new code. Only the first bacteremia episode was included if the patient had repeated blood culture(s) growing the same targeted organisms during the same admission. The culture was excluded if it was sent from the hospital forensic unit, likely a contaminant or a polymicrobial culture with concurrent growth of other gram-negative organisms and other exclusion criteria, as detailed in **Figure 1**. Isolate(s) from the same patient was included if it occurred at a separate admission. The study was approved by the Research Ethics Committee, National University of Malaysia (JEP-2022-187) before the commencement of the study. Informed consent was waived as the study only collected the remnants of the sample sent to and stored in the in-house hospital laboratory as part of routine clinical care. The genotyping analysis only focused on bacteria that were isolated as part of routine hospital laboratory procedures. All patient data were anonymized before the analysis. All the bacteria collected were stocked in 40% glycerol at -80°C.

### 2.2 Antibiotic susceptibility, ESBL phenotype-positive screening (ES) and ESBL phenotype-positive confirmatory test (EC)

Conventional antibiotic susceptibility testing (cAST) was performed using the Kirby–Bauer disk diffusion method. The cAST results were interpreted according to the latest Clinical and Laboratory Standard Institute standards (CLSI) guidelines (Clinical and Laboratory Standards Institute, 2022) and reported in the online microbiological laboratory system. ES was performed using disk diffusion with ceftazidime 30µg (CAZ) and cefotaxime 30µg (CTX) according to CLSI guidelines (Clinical and Laboratory Standards Institute, 2022). Only isolates meeting ES criteria with the diameter of the zone of inhibition ≤ 22mm for CAZ and/or ≤ 27 mm for CTX were subjected to EC test. EC test was done with combination disk diffusion tests, which were performed using CTX, cefotaxime/clavulanic acid (CTX/CLA) (30/10 µg), CAZ, and ceftazidime/clavulanic acid (CAZ/CLA) (30/10 µg). The isolate was reported as an ESBL phenotype-positive when the inhibition zone increases ≥ 5 mm for either combination disk with clavulanate compared to the respective individual disk. The isolates not fulfilling the ES and EC criteria were classified as ESBL phenotype-negative.

### 2.3 β-lactamase genes detection

The β-lactamase genes were identified using multiplex polymerase chain reaction (PCR), performed at UKM Medical Molecular Biology Institute (UMBI) by trained personnel blinded to the organisms’ identity and antibiotic susceptibility profile. Colonies were sub-cultured in 4mL of Muller Hinton Broth (Becton, Dickinson and Company, USA) and incubated overnight at 37°C. Five hundred µLs were then taken from the culture and centrifuged at 12,000g for 5 minutes, and the pellets were re-suspended in 500 µLs of double-distilled water. The solution was then subjected to DNA extraction using a DNeasy Blood & Tissue Kit (Qiagen Inc, USA) according to the manufacturer’s instructions.

The samples were subjected to multiplex PCR using primer sequences and PCR cycling conditions adopted from Ogutu et al. (2015), specific for *bla*_SHV_, *bla*_TEM_, *bla*_CTX-M-1_, *bla*_CTX-M-9_, and *bla*_OXA-1_ group genes as listed in **Table 1**.

**Table 1:** The group-specific primers used in the study, adopted from Ogutu et al. (2015).

Primer name	Sequences	Amplicon size (bp)
M-TEM-F	CATTTCCGTGTCGCCCTTATTC	800
M-TEM-R	CGTTCATCCATAGTTGCCTGAC	
M-SHV-F	AGCCGCTTGAGCAAATTAAAC	713
M-SHV-R	ATCCCGCAGATAAATCACCAC	
M-OXA-1-F	GGCACCAGATTCAACTTTCAAG	564
M-OXA-1-R	GACCCCAAGTTTCCTGTAAGTG	
M-CTX-M-1-F	TTAGGAAGTGTGCCGCTGTA	655
M-CTX-M-1-R	CGGTTTTATCCCCCACAAC	
M-CTXM-9-F	GGTGATGAACGCTTTCCAAT	518
M-CTXM-9-R	TTATCACCYRCAGTCCACGA	

The PCR mixtures were subjected to thermal cycling in a T100 Thermal Cycler (Bio-Rad, U.S.A.) with an initial activation step for 10 mins at 94°C, followed by 30 cycles of denaturation for 30s at 94°C, annealing for 35 s at 61°C and extension for 60s at 72°C, and final extension for 9 mins at 72°C. PCR products were subjected to electrophoresis in 1.5% agarose gel at 80V for 85 mins, and bands were visualized with a ChemiDoc XRS+ (Bio-Rad, U.S.A.).

### 2.4 Predictive value of *bla*_CTX-M_

The performance of *bla*_CTX-M_ in predicting antibiotics non-susceptibility (either intermediate or resistant) or ESBL phenotype-positive isolate was determined by comparing the presence of *bla*_CTX-M_ (either *bla*_CTX-M-1_ or *bla*_CTX-M-9)_ to the phenotypic testing results by cAST. The concordance and discordance (**Supplementary Table 1**) were determined to estimate the Sensitivity (Sen), specificity(Sp), positive (PPV), and negative predictive value (NPV) at a 95% confidence interval, using a diagnostic test evaluation calculator by MedCalc (MedCalc Software Ltd).

### 2.5 Clinical characteristics and outcomes of bacteremia

Patient medical records and the hospital’s online information system were reviewed to extract information on patients’ demographics, comorbidities, systemic organ failure assessment (SOFA) scores, Pitt’s bacteremia score, source of bacteremia, and antibiotics administered. Patients with incomplete admission information were excluded from the analysis. Infections were considered community-acquired if the onset occurred within 48 hours of hospitalization, beyond which they were considered hospital-acquired (Kang et al., 2012). If the infection site was not documented, it was determined according to the definition by the Centers for Disease Control and Prevention/National Healthcare Safety Network (CDC/NHSN, 2022). Immunocompromised status was considered if the patient had a solid organ transplant, hematopoietic stem cell transplant in the past six months prior to the index culture, primary immunodeficiency, human immunodeficiency virus with CD4 count < 200mm^3^, received immunosuppressive therapy or chemotherapy in the past 30 days, neutropenic with an absolute neutrophil count less than 500 cells/mL (CDC, 2003).

Empirical antibiotics referred to the antibiotics administered on blood culture day and before Gram stain results were reported (Carrara et al., 2018). Length of hospital stays and in-hospital mortality within 30 days were calculated from the index culture collection day. Patients with concurrent blood cultures growing other organisms or patients who leave the hospital at their own will were excluded from this analysis.

### 2.6 Statistical analysis

All analyses were done using Statistical Package for the Social Sciences (SPSS), version 29.0 (IBM Corp, Armonk, NY, USA). Descriptive data were described in frequency and percentage. Categorical data were analyzed using the Pearson Chi-Squared or Fisher’s Exact tests where appropriate. The normality of continuous data was tested using the Shapiro-Wilk test. Non-normally distributed variables were presented as median and interquartile range (IQR). The median of continuous variables was compared using the Mann–Whitney test. A p-value of < 0.05 was used as the level of significance.

## 3 Results

A total of 1137 blood cultures from 756 patients were screened during the study period. Only 137 mono-bacterial cultures were eligible for inclusion, consisting of 68 cultures of *E.coli*, 68 cultures of *K. pneumoniae*, and one culture of *K. oxytoca* (**Figure 1**). Nearly one-third (37/137, 27.0%) was ESBL phenotype-positive while two-thirds (100/137, 73.0%) were ESBL phenotype-negative, of which 94 were ES negative and six were EC negative.

### 3.1 Distribution of genotypes and association with ESBL phenotypes

**Figure 2:**
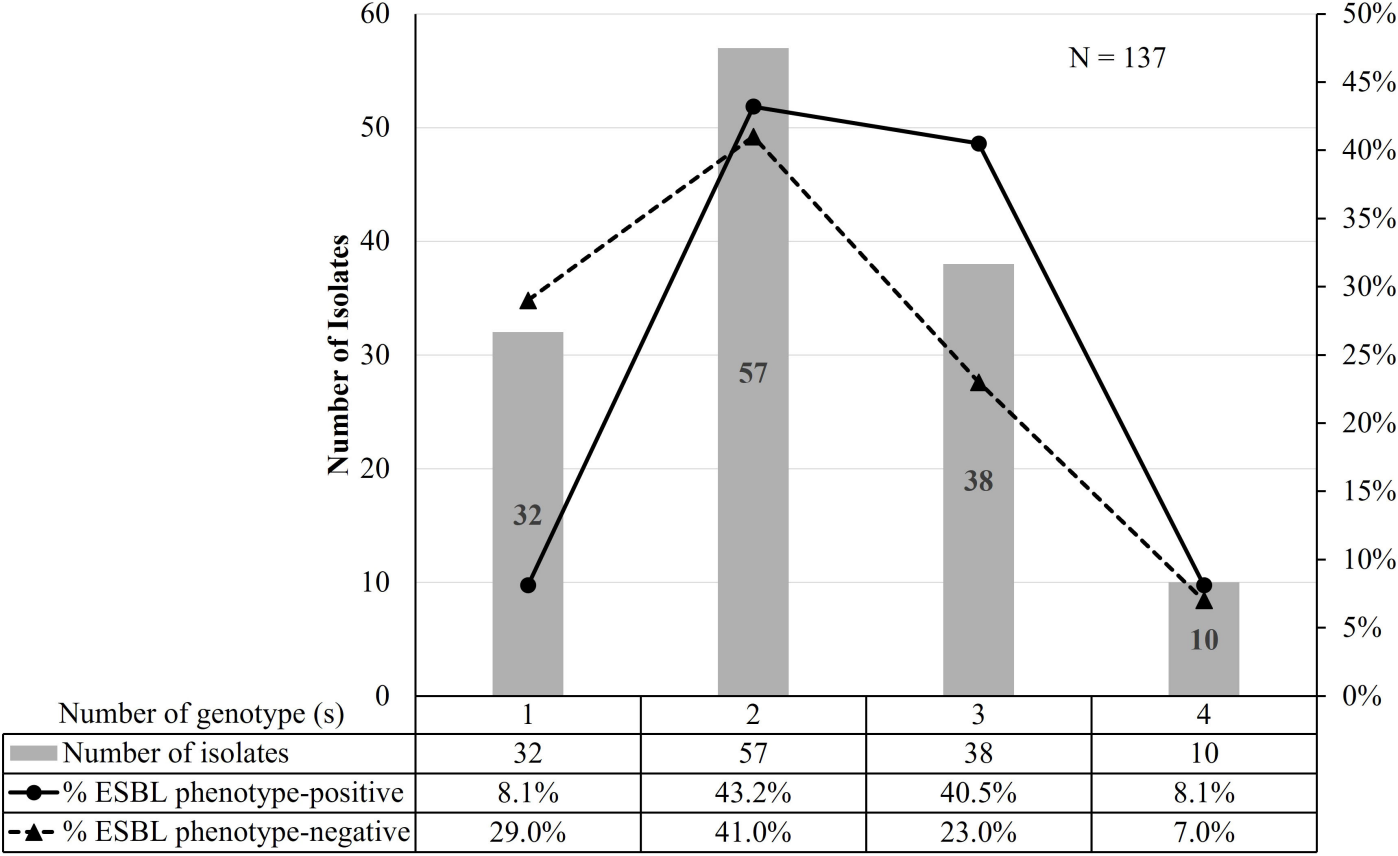
The correlation between the number of genotypes and ESBL phenotypes.

All 137 isolates harbored at least one genotype, including ESBL phenotype-negative isolates. Only 23% (32/137) of isolates carried a single genotype (**Table 2**). The majority of ESBL phenotype-positive isolates harbored at least two genotypes compared to the ESBL phenotype-negative isolates (p = 0.03) (**Figure 2**). We observed that *bla*_CTX-M_ genes were significantly more prevalent in the ESBL phenotype-positive than the ESBL phenotype-negative isolates (P <0.001), which had more *bla*_TEM_ (**Table 3**).

**Table 2:** Distribution of genotypes among ESBL phenotypes.

Genotypes	Overall (N =137)	ESBL phenotype-positive (N = 37)	ESBL phenotype-negative (N = 100)
**Single genotype, n (%)**	32 (23.4)	3 (8.1)	29 (29.0)
*bla*_SHV_	10	1	9
*bla*_TEM_	16	0	16
*bla*_CTX-M-1_	1	1	0
*bla*_CTX-M-9_	2	1	1
*bla*_OXA-1_	3	0	3
**Two genotypes, n (%)**	57 (41.6)	16 (43.2)	41 (41.0)
*bla*_SHV_ + *bla*_TEM_	22	1	21
*bla*_SHV_ + *bla*_CTX-M-1_	7	4	3
*bla*_SHV_ + *bla*_CTX-M-9_	4	2	2
*bla*_SHV_ + *bla*_OXA-1_	1	1	0
*bla*_TEM_ + *bla*_CTX-M-1_	3	2	1
*bla*_TEM_ + *bla*_CTX-M-9_	2	1	1
*bla*_TEM_ + *bla*_OXA-1_	10	1	9
*bla*_CTX-M-1_+ *bla*_CTX-M-9_	0	0	0
*bla*_CTX-M-1_+ *bla*_OXA-1_	6	3	3
*bla*_CTX-M-9_+ *bla*_OXA-1_	2	1	1
**Three genotypes, n (%)**	38 (27.7)	15 (40.5)	23 (23.0)
*bla*_SHV_ + *bla*_TEM_ + *bla*_CTX-M-1_	22	10	12
*bla*_SHV_ + *bla*_TEM_ + *bla*_CTX-M-9_	1	0	1
*bla*_SHV_ + *bla*_TEM_ + *bla*_OXA-1_	4	0	4
*bla*_SHV_ + *bla*_CTX-M-1_+ *bla*_CTX-M-9_	3	0	3
*bla*_SHV_ + *bla*_CTX-M-1_+ *bla*_OXA-1_	2	1	1
*bla*_SHV_ + *bla*_CTX-M-1_+ *bla*_OXA-1_	1	1	0
*bla*_TEM_ + *bla*_CTX-M-1_+ *bla*_CTX-M-9_	0	0	0
*bla*_TEM_ + *bla*_CTX-M-1_+ *bla*_OXA-1_	4	3	1
*bla*_TEM_ + *bla*_CTX-M-9_+ *bla*_OXA-1_	1	0	1
*bla*_CTX-M-1_+ *bla*_CTX-M-9_+ *bla*_OXA-1_	0	0	0
**Four genotypes, n (%)**	10 (7.3)	3 (8.1)	7 (7.0)
*bla*_SHV_ + *bla*_TEM_ + *bla*_CTX-M-1_+ *bla*_CTX-M-9_	6	0	6
*bla*_SHV_ + *bla*_TEM_ + *bla*_CTX-M-1_+ *bla*_OXA-1_	3	2	1
*bla*_SHV_ + *bla*_TEM_ + *bla*_CTX-M-9_+ *bla*_OXA-1_	1	1	0

**Table 3:** Association of genotypes and ESBL phenotypes in tested isolates.

Gene (s)	Overall^§^ (N =137)	*E.coli* (N =68)	*K.pneumoniae* (N =68)	ESBL phenotypes		p*
				Positive (N = 37)	Negative (N=100)	
*bla*_CTX-M_, n (%)	71 (51.8) ^	29 (42.6)	41 (60.3)	33 (89.2)	38 (38.0)	<0.001^a^
*bla*_SHV_, n (%)	87 (63.5)	21 (30.9)	65 (95.6)	24 (64.9)	63 (63.0)	0.840 ^a^
*bla*_TEM_, n (%)	95 (69.3)	51 (75.0)	43 (63.2)	21 (56.8)	74 (74.0)	0.052 ^a^
*bla*_OXA-1_, n (%)	38 (27.7)	31 (45.6)	7 (10.3)	14 (37.8)	24 (24.0)	0.108 ^a^

^§^Overall consisted of 68 isolates of *E. coli*, 68 isolates of *K. pneumoniae*, and one isolate of *K. oxytoca*.

^*bla*_CTX-M-1_ (n = 57), *bla*_CTX-M-9_ (n = 38), *bla*_CTX-M-1_ & *bla*_CTX-M-9_ (n = 9);

a Pearson Chi-square.

*p < 0.05 indicates statistically significant.

### 3.2 Antibiotic susceptibilities of isolates harboring *bla*_CTX-M_

At least half of the isolates harboring *bla*_CTX-M_ retained susceptibility to β-lactam antibiotics, including nine *E.coli* isolates (12.7%) that were ampicillin susceptible (**Table 4**). Nearly half were resistant to 3GC and cefepime, and were confirmed to be ESBL phenotype-positive. In addition, less than one-third were resistant to penicillin/β-lactamase inhibitors (PCNBLI). Besides, piperacillin/tazobactam susceptibility remained in nearly three-quarters of isolates despite being ESBL phenotype-positive and *bla*_CTX-M_ positive.

**Table 4:** The antibiogram of the tested isolates harboring *bla*_CTX-M_.

Antibiotics	*bla*_CTX-M_-positive All (N=71)						*bla*_CTX-M_ -positive ESBL phenotype-positive (N=33)						*bla*_CTX-M_ -positive ESBL phenotype-negative (N=38)					
	S		I		R		S		I		R		S		I		R	
	n	%	n	%	n	%	n	%	n	%	n	%	n	%	n	%	n	%
Ampicillin	9	12.7	1	1.4	61	85.9	0	0.0	1	3.0	32	97.0	9	23.7	0	0.0	29	76.3
Ampicillin/Sulbactam^§^	45	64.3	6	8.6	19	27.1	11	34.4	5	15.6	16	50.0	34	89.5	1	2.6	3	7.9
Amoxicillin/Clavulanate^§^	49	71.0	11	15.9	9	13.0	14	43.8	11	34.4	7	21.9	35	94.6	0	0.0	2	5.4
Piperacillin/Tazobactam	55	77.5	7	9.9	9	12.7	24	72.7	3	9.1	6	18.2	31	81.6	4	10.5	3	7.9
Cefuroxime	34	47.9	2	2.8	35	49.3	0	0.0	1	3.0	32	97.0	34	89.5	1	2.6	3	7.9
Cefotaxime	37	52.1	1	1.4	33	46.5	0	0.0	1	3.0	32	97.0	37	97.4	0	0.0	1	2.6
Ceftriaxone	39	54.9	0	0.0	32	45.1	1	3.0	0	0.0	32	97.0	38	100.0	0	0.0	0	0.0
Ceftazidime	43	60.6	4	5.6	24	33.8	6	18.2	4	12.1	23	69.7	37	97.4	0	0.0	1	2.6
Cefepime	41	57.7	6	8.5	24	33.8	3	9.1	6	18.2	24	72.7	38	100.0	0	0.0	0	0.0
Ertapenem	70	100.0	0	0.0	0	0.0	33	100.0	0	0.0	0	0.0	37	100.0	0	0.0	0	0.0
Meropenem	71	100.0	0	0.0	0	0.0	33	100.0	0	0.0	0	0.0	38	100.0	0	0.0	0	0.0
Imipenem	71	100.0	0	0.0	0	0.0	33	100.0	0	0.0	0	0.0	38	100.0	0	0.0	0	0.0

^§^Antibiotic susceptibility was not tested for one isolate for Ampicillin/Sulbactam and two isolates for Amoxicillin/Clavulanate.

S, susceptible.

I, Intermediate susceptible.

R, Resistance.

### 3.3 Predictive values of *bla*_CTX-M_

Referring to the antibiogram (**Table 4**), 38 of 100 ESBL phenotype-negative isolates were *bla*_CTX-M_ positive, resulting in a Sp of only 62% and Sen of 89%. Likewise, the Sp of *bla*_CTX-M_ to predict the 3GC and cefepime resistance ranged from 59.34% to 60.58%, though the Sen was from 71.79% to 90.91% (**Table 5**).

**Table 5:** Predictive value of *bla*_CTX-M_ for ESBL phenotype-positive isolates and cephalosporins resistance.

	Sen % (95% CI)	Sp % (95% CI)	PPV % (95% CI)	NPV % (95% CI)
ESBL phenotype-positive	89.19 (74.58 to 96.97)	62.00 (51.75 to 71.52)	46.47 (39.75 to 53.32)	93.94 (85.85 to 97.54)
Cefotaxime	73.91 (58.87 to 85.73)	59.34 (48.53 to 69.52)	47.91 (40.48 to 55.44)	81.80 (72.86 to 88.27)
Ceftriaxone	80.00 (64.35 to 90.95)	59.79 (49.35 to 69.63)	45.07 (38.09 to 52.25)	87.88 (79.25 to 93.23)
Ceftazidime	71.79 (55.13 to 85)	56.12 (45.73 to 66.13)	39.48 (32.62 to 46.77)	83.31 (74.60 to 89.46)
Cefepime	90.91 (75.67 to 98.08)	60.58 (50.51 to 70.02)	42.27 (36.05 to 48.75)	95.45 (87.59 to 98.42)

CI, confidence interval; Sen, sensitivity; Sp, specificity; PPV, positive predictive value; NPV, negative predictive value.

### 3.4 Clinical characteristics of bacteremia associated with ESBL phenotype-negative isolates

Ninety-eight patients with ESBL phenotype-negative bacteremia were eligible for clinical analysis (**Figure 1**). We observed an equal distribution of elderly and younger patients (49/98, 50%); slightly less than half were male (**Table 6**). Malay ethnicity was the majority, followed by Chinese and Indian. More patients in the *bla*_CTX-M_ group received antibiotics in the past 90 days prior to bacteremia episodes. Patients had a median of four comorbidities and were comparable between groups. However, the non-*bla*_CTX-M_ group had a remarkably higher fraction of diabetics with end-organ damage (with retinopathy, neuropathy or nephropathy) (10.8% vs 29.5%, p = 0.032) and renal failure (0% vs 11.5%, p = 0.043). More than half of the population were medical patients, and one-third were surgical patients. On the day of infection onset, less than one-tenth were admitted to intensive care or high-dependency units. Nevertheless, no distinct dissimilarity between groups was observed for hospital- and community-acquired bacteremia frequencies. The infection foci were also comparable, except for a significantly higher fraction of the gastrointestinal site in the non-*bla*_CTX-M_ group (44.3% vs 24.3%, p = 0.047).

**Table 6:** Patient characteristics of bacteremia by ESBL phenotype-negative isolates.

	Overall (N = 98)	bla_CTX-M_ (N = 37)	Non-bla_CTX-M_ (N = 61)	p*
Male, n (%)	44 (44.9)	16 (43.2)	28 (45.9)	0.798 ^a^
Age [year], median (IQR)	65.0 (53.75-76.00)	63.0 (55.0-73.5)	66.0 (53.0-79.0)	0.741 ^b^
Weight [kg], median (IQR)	70.0 (58.15-80.0)	70.0 (60.0-74.0)	70.0 (56.35-80.0)	0.811 ^b^
Race, n (%)				0.546 ^c^
Malay	51 (52.0)	20 (54.1)	31 (50.8)	
Chinese	31 (31.6)	11 (29.7)	20 (32.8)	
Indian	12 (12.2)	4 (10.8)	8 (13.1)	
Others	4 (4.0)	2 (5.4)	2 (3.2)	
Antibiotics history, n (%)	37 (37.8)	10 (27.0)	27 (44.30)	0.088 ^a^
CCI^^^, median (IQR)	4.0 (3.0-6.0)	4.0 (2.0-6.0)	4.0 (3.0-7.0)	0.135 ^c^
Comorbidities, n (%)				
Myocardial infarction,	10 (27.0)	10 (27.0)	17 (27.9)	0.928 ^a^
Cardiac failure	11 (11.2)	3 (8.1)	8 (13.1)	0.527 ^c^
Peripheral vascular disease	2 (2.0)	0 (0)	2 (3.3)	0.525 ^c^
Diabetes with end organ damage^	22 (22.4)	4 (10.8)	18 (29.5)	0.032 ^a^
Liver cirrhosis	1 (1.0)	0 (0)	1 (1.6)	1.000 ^c^
Renal insufficiency	7 (7.1)	0 (0)	7 (11.5)	0.043 ^c^
Solid tumor	18 (18.4)	7 (18.9)	11 (18.0)	0.913 ^a^
Leukemia	2 (2.0)	1 (2.7)	1 (1.6)	1.000 ^c^
Lymphoma	2 (2.0)	1 (2.7)	1 (1.6)	1.000 ^c^
Immunocompromised	9 (9.2)	4 (10.8)	5 (8.2)	0.726 ^c^
Disciplines of care, n (%)				0.557 ^c^
Medical	57 (58.2)	24 (64.9)	33 (54.1)	
Surgical	35 (35.7)	11 (29.7)	24 (39.3)	
Oncology	4 (4.1)	2 (5.4)	2 (3.3)	
Gynecology	2 (2.0)	0 (0)	2 (3.3)	
Admission to intensive care/high dependency unit, n (%)	6 (6.1)	1 (2.7)	5 (8.2)	0.404 ^c^
Bacteremia settings, n (%)				
Community	36 (36.7)	16 (43.2)	20 (32.8)	0.574 ^a^
Healthcare-associated	48 (49.0)	16 (43.2)	32 (52.5)	
Hospital-acquired	14 (14.3)	5 (13.5)	9 (14.8)	
Infection site(s) ^¥^, n (%)				
Respiratory	31 (31.6)	13 (35.1)	18 (29.5)	0.561 ^a^
Gastrointestinal	36 (36.7)	9 (24.3)	27 (44.3)	0.047 ^a^
Genitourinary	31 (31.6)	14 (37.8)	17 (27.9)	0.304 ^a^
Skin & Soft Tissue	1 (1.0)	0 (0.0)	1 (1.6)	1.000 ^c^
Bone & Joint	2 (2.0)	2 (5.4)	0 (0.0)	1.000 ^c^
Primary bacteremia	3 (3.1)	2 (5.4)	1 (1.6)	0.555 ^c^
Bacteria, n (%)				
*E.coli*	49 (50.0)	11 (29.7)	38 (62.3)	
*K.pneumoniae*	49 (50.0)	26 (70.3)	23 (37.7)	0.002 ^a^

IQR, interquartile range; CCI^, Charlson Comorbidity Index; Pitt, Pitt bacteremia score on blood culture day.

^¥^Sites of infections are not mutually exclusive.

^Diabetes with end organ damage refers to retinopathy, neuropathy or nephropathy attributable to diabetes.

a Pearson Chi-square.

b Mann-Whitney.

c Fisher’s exact test; *p < 0.05 indicates statistically significant.

### 3.5 Bacteremia severity, clinical outcomes, and association with empirical antibiotics

Most patients were prescribed empirical PCNBLI or 3GC, and only one was given empirical meropenem in each group (**Table 7**). Overall, there was no remarkable between-group difference observed in the bacteremia severity at onset, apart from fever recorded as part of Pitt’s bacteremia scores, with body temperature trending towards higher median readings in the *bla*_CTX-M_ group (38.2°C vs 38.6°C, p = 0.054) (**Table 8**). One-third of the patients in each group had septic shock. Both groups were also similar in the mortality rate (16%), with hospital stays at a median of ten days. More patients in the non-*bla*_CTX-M_ group received carbapenems within seven days since blood culture day, but three of them died during admission. Nonetheless, further analysis of each empirical antibiotics class found that neither the listed genotypes nor the carriage of two genotypes or more differed significantly in mortality rate (**Table 9**).

**Table 7:** Antibiotics initiated in bacteremia by ESBL phenotype-negative isolates.

	Overall (N = 98)	*bla*_CTX-M_ (N = 37)	Non-*bla*_CTX-M_ (N = 61)	p*
Empirical antibiotics, n (%)				
Aminopenicillins/inhibitors^#,§^	38 (38.8)	11 (29.7)	27 (44.3)	0.152 ^a^
Piperacillin/Tazobactam^§^	33 (33.7)	15 (40.5)	18 (29.5)	0.263 ^a^
Second-generation cephalosporin	1 (1.0)	0 (0)	1 (1.6)	
Third-generation cephalosporins^	24 (24.5)	10 (27.0)	14 (23.0)	0.649 ^a^
Meropenem	2 (2.0)	1 (2.7)	1 (1.6)	
Receipt of Carbapenem within seven days since blood culture day, n (%)	9 (9.2)	2 (5.4)	7 (11.5)	0.476 ^b^

^#^Aminopenicillins/inhibitors: Amoxicillin/clavulanic acid or Ampicillin/sulbactam.

^§^Aminopenicillins/inhibitors and piperacillin/tazobactam are referred to as penicillin/beta-lactamase inhibitors (PCNBLI).

^Third-generation cephalosporins refer to ceftriaxone, cefotaxime or ceftazidime.

a Pearson Chi-square.

b Fisher’s exact test.

*p < 0.05 indicates statistically significant.

**Table 8:** Severity & clinical outcome of bacteremia by ESBL phenotype-negative isolates.

	Overall	*bla*_CTX-M_	Non-*bla*_CTX-M_	p*
Severity, N	98	37	61	
Septic shock, n (%)	30 (30.6)	10 (27.0)	20 (32.8)	0.549 ^a^
SOFA^#^ at onset, median (IQR)	4.0 (2.0-6.0)	4.0 (2.0-6.5)	4.0 (2.0-6.0)	0.865 ^b^
Pitt^^^ score, median (IQR)	1.0 (0.0-2.0)	1.0 (0.0-2.0)	1.0 (0.0-2.5)	0.906 ^b^
Pitt^^^ >/= 4, n (%)	10 (10.2)	3 (8.1)	7 (11.5)	0.738 ^c^
Temperature,°C, median (IQR)	38.5 (37.8-39.2)	38.6 (38.2 - 39.3)	38.2 (37.0 - 39.2)	0.054 ^b^
Outcomes, N	93	37	56	
Hospital discharge, n (%)	78 (83.9)	31 (83.8)	47 (83.9)	0.985 ^a^
Length of stay, median (IQR)	10.0 (7.0-13.3)	10.0 (7.0-16.0)	10.0 (7.0-13.0)	0.992 ^b^
In-hospital Mortality^§^, n (%)	15 (16.1)	6 (16.2)	9 (16.1)	0.985 ^a^
Mortality among patients receiving carbapenems within seven days since blood culture day^^, n (%)	3 (33.3)	0 (0.0)	3 (42.9)	0.500 ^c^

IQR, interquartile range.

^#^SOFA, Sequential organ failure assessment score on blood culture day.

^Pitt, Pitt bacteremia score on blood culture day.

^§^In-hospital mortality at 30 days.

^^Total 9 patients: 2 patients from *bla*_CTX-M_ and 7 patients from Non-*bla*_CTX-M_ group.

a Pearson chi-square.

b Mann-Whitney.

c Fisher’s exact test.

*p < 0.05 indicates statistically significant.

**Table 9:** Genotype and 30-day in-hospital mortality according to empirical antibiotics group initiated in bacteremia by ESBL phenotype-negative isolates.

Presence of genotype	30-day in-hospital mortality, n (%)					
	Empirical piperacillin-tazobactam (N = 33)	p*	Empirical aminopenicillin(s)/inhibitor(s) ^#^ (N = 35)	p*	Empirical third-generation cephalosporins^ (N = 23)	p*
*bla*_CTX-M_	2/15 (13.3)	0.413^a^	2/11 (18.2)	0.640 ^a^	2/10 (20.0)	0.560 ^a^
*bla*_SHV_	7/24 (29.2)	0.149^a^	3/18 (16.7)	1.000 ^a^	3/14 (21.4)	0.253 ^a^
*bla*_TEM_	6/25 (24.0)	0.652^a^	3/25 (12.0)	0.610 ^a^	1/16 (6.30)	0.209 ^a^
*bla*_OXA-1_	0/6 (0)	0.301^a^	2/9 (22.2)	0.586 ^a^	1/7 (14.3)	0.586 ^a^
Two or more genotypes	6/27 (22.2)	1.000^a^	2/19 (10.5)	1.000 ^a^	3/18 (13.0)	1.000 ^a^

a Fisher’s exact.

*p < 0.05 indicates statistically significant.

^#^aminopenicillins/inhibitors: Amoxicillin/clavulanic acid or Ampicillin/sulbactam.

^third-generation cephalosporins refers to ceftriaxone, cefotaxime or ceftazidime.

## 4 Discussion

The diagnostic approach for identifying the ESBL-producing organism has been a subject of contention (Mathers and Lewis, 2021; Villegas et al., 2021), for which Tamma and Humphries (2021) advocate for the inclusion of molecular detection for ESBL genes in susceptibility tests. Nevertheless, apprehending the resistance epidemiology from the published studies, particularly in gram-negative bacteria, can be an endeavor. There is great diversity in the assessment approaches regarding inclusion criteria, sample origin, diagnostic methods, and the ESBL producer(s) definition (Ling et al., 2021; Tamma et al., 2021). Furthermore, most of the prevalence studies of ESBL genotypes were assessed in ESBL phenotypes-positive or 3GC-resistant strains isolated from various body sites not limited to blood.

Several studies from the United States (US) (Mushtaq et al., 2022), Europe, Asia (Wu et al., 2011; Vlieghe et al., 2015; Heng et al., 2018; Ling et al., 2021; Son et al., 2021; Salawudeen et al., 2023) and other regions (Ramadan et al., 2019; Legese et al., 2022; Lubwama et al., 2024) unanimously reported *bla*_CTX-M_ as the predominating ESBL genotype with detection rate as high as 90% of the isolates. Our findings were consistent with a significantly higher proportion of *bla*_CTX-M_ (89.2%) in the ESBL-positive phenotypes than in the negative phenotype. Besides, we noted that the ESBL phenotype-positive isolates frequently harbored at least two or more genotypes, as observed in previous studies (Heng et al., 2018; Ramadan et al., 2019; Ngoi et al., 2021). *bla*_CTX-M_ is commonly used as an ESBL marker (De Angelis et al., 2020b). Hence, we concentrated on evaluating the correlation and predictability of *bla*_CTX-M_ to ESBL phenotypes and the clinical significance in bacteremia by isolates that were *bla*_CTX-M_ -positive but ESBL phenotype-negative.

Limited studies include susceptible strains in the ESBL genotype investigation. At the start of the millennium, Lim et al. investigated ESBL genotypes among all clinical isolates of *Enterobacteriaceae* from various body sites. Across the phenotypes, *bla*_SHV_, *bla*_TEM_, *bla*_CTX-M_, and *bla*_OXA_ were detected, with the most prevalent genotypes as *bla*_TEM_ (35/47, 74.5%) in *E.coli* (Lim et al., 2009a) and *bla*_SHV_ (46/51, 90.2%) in *K.pneumoniae* (Lim et al., 2009b), whereas *bla*_CTX-M_ was detected in only 17% (8/47) of *E.coli* and 37.3% (19/51) of *K.pneumoniae*. While *bla*_SHV_ and *bla*_TEM_ distribution in our study were consistent with Lim et al., we detected almost twice the portion of *bla*_CTX-M_ in the tested isolates of *E. coli* and *K. pneumoniae*. This aligns with the increasing spread of *bla*_CTX-M_ over the years following the global phenomenon (Bevan et al., 2017). Nevertheless, the distribution could vary according to the origin of the isolates, as noted in the global investigation by Yu et al. (2024), which identified *bla*_CTX-M_ in 61.4% (51/83) of *E.coli* isolated from blood, higher than those from urine (42.9%, 69/161) and feces (17%, 45/264). Son et al. (2021) also found as high as 70% *bla*_CTX-M_ in 115 isolates from blood in Vietnam. In contrast, a large-scale epidemiological study in the US found the prevalence of *bla*_CTX-M_ within the collective population of *E.coli* and *K.pneumoniae* as 15.8% (433/2746), ranging from 5% to 26% by state (Tamma et al., 2021). A subsequent study by Mushtaq et al. (2022) in US noted a similar prevalence of *bla*_CTX-M_, correlating well with the resistance profiles of over 200 *bla*_CTX-M_ positive organisms, but the three discordant ceftriaxone susceptible organisms raised concern about the potential implications.

To the best of our knowledge, the assessment of genotypes among susceptible or ESBL phenotype-negative clinical isolates in the human sector were mainly commenced in Asia countries. Zhang et al. (2018) in China found the presence of ESBL genes among cefotaxime or ceftazidime susceptible strains of *K.pneumoniae* and detected *bla*_SHV_ and *bla*_CTX-M_ in 3.0% (6/202) and 5.9% (12/202) of the strains, respectively. The study by Ogutu et al. (2015), from whom we adopted the multiplex PCR method, identified *bla*_CTX-M-1_ and/or *bla*_CTX-M-9_ in 40.4% of their studied strains that were mostly 3GC resistant. In contrast, Son et al. (2021) in Vietnam reported a higher degree of discordant results, with one-third of the *bla*_CTX-M_ harboring *E.coli* isolates being phenotypically ESBL-negative (22/80, 27.5%). In conjunction with this, our study recorded an even higher discrepancy between ESBL genotypes and phenotypes, as nearly half of the *bla*_CTX-M_ harboring strains were 3GC susceptible or ESBL phenotype-negative (**Table 4**). The discrepancies in our study also render the detection of *bla*_CTX-M_ by PCR low Sp and PPV values for predicting ESBL phenotype-positive and 3GC resistance compared to the results from China (Ogutu et al., 2015) and Vietnam (Son et al., 2021). We suspect the difference in the ESBL genotype-phenotype concordance implies variations in ESBL phenotypic expression across geographical locations.

Carriage of ESBL genes by susceptible strains raises concerns about the possible clinical implications attributed to the presence of *bla*_CTX-M_ (Tamma and Humphries, 2021; Mushtaq et al., 2022). Therefore, we further explored patient-level information and clinical outcomes among ESBL phenotype-negatives harboring *bla*_CTX-M_ versus those without *bla*_CTX-M_ but were *bla*_TEM_/*bla*_SHV_/*bla*_OXA-1_ positive. We observe that our patient characteristics largely resemble the population studied in Vietnam (Son et al., 2021), except for a higher prevalence of bacteremia from respiratory sources. In our patients, the lack of association between patient clinical variables and *bla*_CTX-M_ isolate infection is similar to previous studies on phenotypically resistant isolates (Wu et al., 2011; Heng et al., 2018). Moreover, the observation of significantly more renal-impaired patients in the non-*bla*_CTX-M_ group conforms with previous findings that renal morbidities were an independent predictor of non-*bla*_CTX-M_ carriage (Wu et al., 2011; Logan et al., 2021). Regarding the bacteremia source, the intra-abdominal foci were less likely to have ESBL phenotype-negative isolates harboring *bla*_CTX-M_. Contrastingly, Wu et al. (2011) did not find an association between infection foci and *bla*_CTX-M_ positivity among phenotypic-resistant bacteria.

We did not find that bacteremia by ESBL phenotype-negative isolates harboring *bla*_CTX-M_ genes has worse infection severity than the non-*bla*_CTX-M_ group, as assessed by SOFA and Pitt scores. On the contrary, the patients in the non-*bla*_CTX-M_ group in the study by Wu et al. (2011) were more ill. Nevertheless, our findings are consistent with a systematic review (Ling et al., 2021) that genotypically confirmed ESBL producers were not associated with a higher frequency of septic shock. The mortality rate in the current study is within the range reported with *Enterobacteriaceae* bacteremia (Heng et al., 2018; de Lastours et al., 2020; Abubakar et al., 2022; Handal et al., 2024). The subpopulation analysis by Son et al. (2021) found that *bla*_CTX-M_ presence in ESBL-negative phenotypes had higher mortality (8% vs 27%, p = 0.07) and twice prolonged hospitalization (27.4 ± 24.5 vs 14.4 ± 7.5, p = 0.014) than those without the genotype. In contrast, we do not find the same detrimental effect of the unexpressed *bla*_CTX-M_ genes on in-hospital mortality and hospital stay. On a related note, the precedent studies that analyzed 3GC-resistant organisms also found comparable mortality between genotypes (Wu et al., 2011; Heng et al., 2018). Likewise, in the recent multicenter observational study by Hareza et al. (2023) recruiting the ceftriaxone-resistant Enterobacterales, the mortality rate between 370 *bla*_CTX-M_ and 33 non-*bla*_CTX-M_ carrying isolates was not significantly different (OR 0.99; 95% CI,.87–1.11). Although two systematic reviews converged on a trend towards worsening outcomes, both could not establish a significant association between phenotypic or genotypic ESBL and mortality rates or hospital stays in bacteremia, owing to the heterogeneity in the studies’ designs (Shamsrizi et al., 2020; Ling et al., 2021). Consequently, the in-hospital death in the current study is likely explained by the comparable host characteristics, morbidities, and mortality predictors (Paterson et al., 2004; Shankar-Hari et al., 2016; Singer et al., 2016).

One of the questions raised in our study was the adequacy of treatment options for bacteremia caused by genotypic positive ESBL phenotype-negative isolates (Mushtaq et al., 2022). A study in Taiwan observed a 100% mortality rate with cefepime therapy in ten cefepime-susceptible ESBL-producing isolates but none in non-ESBL producers (P < 0.001) (Lee et al., 2015). *Post hoc* analysis of the MERINO trial recommended carbapenem over piperacillin/tazobactam for the gram-negative bacteremia co-harboring *bla*_OXA_ (Henderson et al., 2021). On the other hand, the exploratory analysis by Hareza et al. (2023) among non-*bla*_CTX-M_ carrying ceftriaxone-resistant Enterobacterales reported a better survival rate among patients receiving meropenem than those receiving piperacillin/tazobactam (18/21, 86% versus 0/5). These studies suggested the potential deleterious effect the genotype detected on antibiotics efficacy. Therefore, we probed into the empirical antibiotics as the appropriateness of the antibiotics initiated upon blood culture collection was associated with fatality (Vazquez-Guillamet et al., 2014; Bonine et al., 2019). However, within each empirical non-carbapenem antibiotic class prescribed, no significant difference in mortality was observed between genotypes in ESBL phenotype-negative bacteremia, including the comparison with *bla*_CTX-M_ (**Table 9**). Analysis of definitive therapy was not done as most were continued non-carbapenem antibiotics. Only nine patients were given carbapenem within seven days past blood culture collection, and three were deceased, albeit all were from the non-*bla*_CTX-M_ group; the number was too small to make any proposition. Therefore, we could not deduce the need for carbapenem in ESBL phenotype-negative bacteremia despite the detection of *bla*_CTX-M_, as suggested by Tamma et al. (2021). The current findings are essential when considering molecular testing in clinical practice and antibiotic prescribing when encountering similar phenotype-genotype discrepancies in our settings.

The detected genes in ESBL phenotype-negative strains reveal the existence of silent genes (Deekshit and Srikumar, 2022). These were often discovered in clinical settings, implying the impending spread of resistance in hospitals. Such phenomenon was also observed in other organisms, such as IMP gene in 25% of carbapenem susceptible *K.pneumoniae* and *bla*_OXA-23_ in 16.13% of imipenem susceptible *Acinetobacter baumannii*. The genes stayed silent based on the notion of fitness conservation for survival through several mechanisms outlined by Stasiak et al. (2021): mutational conversion into non-functional genes; defect in the gene regulatory system comprised of errors in gene expression regulator, loss of essential gene clusters, deactivation due to integrons structure modification and xenogeneic silencing protein activity; laboratory conditions not representing the environment responsible for the phenotypic presentation. Hence, the presented *bla*_CTX-M_ positive ESBL phenotype negative might be attributed to the variants of diminished protein activity as a result of compromised fitness due to mutational change, or the genes were not quantitatively sufficient to express phenotypic resistance (Mushtaq et al., 2022). Conversely, the cryptic genes carried on the plasmids or chromosomes can be disseminated through horizontal gene transfer and be activated in other pathogenic bacteria (Zhang et al., 2018; Deekshit and Srikumar, 2022). Furthermore, the silencing is not permanent and could be reversible to activate expression under suitable conditions such as antibiotic pressure (Stasiak et al., 2021). Therefore, the unexpressed genes could represent bacteria’s resistance capability and pose a therapeutic challenge and public health concern (Zhang et al., 2018). It is noteworthy in our study that ESBL phenotype-negative isolates also carried multiple genotypes, albeit to a lesser extent than the ESBL-positive phenotype isolates. From an antimicrobial stewardship perspective, narrow-spectrum antibiotics should be used for susceptible organisms, as most, if not all, organisms harboring the resistant gene(s) could undergo mutation and potentially transfer resistance to other non-clinical targeted bacteria bystanders under antibiotic pressure (Alm and Lahiri, 2020).

The reference phenotypic method used in this study was the disk diffusion of cefotaxime or ceftazidime as the proxy and combined disk diffusion as a confirmatory test, which was the recommended phenotypic method by Clinical and Laboratory Standards Institute (2022) for ESBL production, with high sensitivity and specificity (Willems et al., 2013). Furthermore, the disk diffusion of cefotaxime and ceftazidime was found by Tenover et al. (2020) to be highly agreeable (97.7% and 91.7%, respectively) with the reference broth microdilution testing using MicroScan Walkaway platform. Although the ESBL confirmatory test could be rendered false negative due to co-existing AmpC over-expression, this was not investigated in the current study, and only six isolates were grouped into the ESBL phenotype-negative population due to a negative confirmatory test.

Our study adds to the limited information available on the genotypic distribution in all strains, not only among the resistant but also the susceptible or ESBL phenotype-negative *Enterobacteriaceae*, which were often unexplored in most studies. Moreover, we provide a better understanding of the genotypic prevalence among the clinical isolates of invasive *E.coli* and *K.pneumoniae* causing bacteremia. Furthermore, the current study is one of the few that incorporate patient-level clinical information and antibiotics use to elucidate the clinical value of ESBL genotypic testing and its application in Southeast Asia or resource-constrained developing countries.

There are several limitations in our study. Firstly, only *bla* groups were detected using PCR. The composition of *bla*_SHV_ and *bla*_TEM_ ESBL variants was not determined. Hence, the detected *bla*_SHV,_ *bla*_TEM_, and *bla*_OXA-1_ could be those of narrow-spectrum β-lactamases, such as *bla*_TEM-1_ in *E.coli* or *bla*_SHV-1_, which *K.pneumoniae* inherently carries (Paterson and Bonomo, 2005). Additionally, the primers for *bla*_CTX-M_ group genes could have increased the detection sensitivity. The *bla*_CTX-M-1_ and/or *bla*_CTX-M-9_ group genes were included as the targets for *bla*_CTX-M_ in this study as they were the majority detected among the 3GC-resistant strains in the earlier local study in the same hospital by Othman et al. (2016). Complete genome analysis was not performed, though similar insignificant results might be expected, as reported by recent studies that examined genome mutations and virulence factors (Heng et al., 2018; de Lastours et al., 2020). Secondly, we did not have minimum inhibitory concentration (MIC) data to observe for any variation correlating with the detected genotype(s), as MIC testing was unavailable during the present study period. Lastly, the cohorts included were from a single tertiary center. Hence, the current findings might not be generalizable to other settings or regions. Future studies using different phenotypic tests, such as broth microdilution and molecular tests of ESBL variants, are proposed to validate the current findings.

## 5 Conclusions

The current study provides insight into the carriage of genes in *E.coli* and *Klebsiella species* clinical isolates, including *bla*_CTX-M_ genotypes in antibiotic-susceptible strains from a Malaysian tertiary center, in a one-year duration. The ESBL encoding *bla*_CTX-M_ genotype presented substantially beyond one-third of the ESBL phenotype-negative or 3GC susceptible *E.coli* and *K.pneumoniae* strains isolated from bloodstream infection. This resulted in the detection of *bla*_CTX-M_ by PCR having suboptimal performance in predicting ESBL phenotypes or antibiotic susceptibilities in our study. Further evaluation of bacteremia by ESBL phenotype-negative isolates did not find that *bla*_CTX-M_ carriage had clinical significance on patient outcomes compared to non-*bla*_CTX-M_. The same was observed with the subgroup analysis of empirical non-carbapenem antibiotics. These findings are essential when considering molecular ESBL testing in clinical practice in our settings. Nevertheless, the potential implications of ESBL genotype-positive but phenotype-negative isolates for the AMR spread deserve further investigation.

## Data Availability

The original contributions presented in the study are included in the article/**Supplementary Material**. Further inquiries can be directed to the corresponding author.

## Supplementary material

Supplementary Table 1 Definition of true positive, true negative, false positive, and false negative results for *bla*_CTX-M_ with reference to ESBL phenotypes and antibiotic susceptibilities.
