# Supplementary material for: Prevalence and clinical significance of the genotypic carriage among ESBL phenotype-negative Escherichia coli and Klebsiella pneumoniae clinical isolates in bacteremia: a study in a Malaysian tertiary center
Source: Front Cell Infect Microbiol. 2024 Oct 24;14:1429830. doi: 10.3389/fcimb.2024.1429830 (PMC11540778; doi:10.3389/fcimb.2024.1429830)
Supplement: Supplementary Table 1 — Definition of true positive, true negative, false positive, and false negative results for bla CTX-M with reference to ESBL phenotypes and antibiotic susceptibilities. [file Table1.pdf]

Table S1 Definition of true positive, true negative, false positive, and false negative results for *bla*$_{CTX-M}$ with reference to ESBL phenotypes and antibiotic susceptibilities

bla$_{CTX-M}$	ESBL phenotypes		Antibiotic susceptibility by cAST	
	Positive	Negative	Not susceptible*	Susceptible
Presence	True positive	False positive	True positive	False positive
Absence	False negative	True negative	False negative	True negative

* Isolate was intermediate susceptible or resistant.
ESBL, Extended-spectrum beta-lactamases; cAST, Conventional antibiotic susceptibility testings.

The concordance and discordance (Table S1) were determined to estimate the Sensitivity(Sen), specificity(Sp), positive (PPV), and negative predictive value (NPV) at a 95% confidence interval, using a diagnostic test evaluation calculator by MedCalc (MedCalc Software Ltd.).
